# Health-related quality of life of young academics: A cross-sectional survey of universities in Wuhan, China

**DOI:** 10.3389/fpsyg.2022.996219

**Published:** 2022-11-10

**Authors:** Qiang Yao, Fei Yang, Hanxuan Li, Kaiyue Tang, Chaojie Liu

**Affiliations:** ^1^Centre for Social Security Studies, Wuhan University, Wuhan, Hubei, China; ^2^School of Political Science and Public Administration, Wuhan University, Wuhan, Hubei, China; ^3^School of Psychology and Public Health, La Trobe University, Melbourne, VIC, Australia

**Keywords:** EQ-5D-5L, China, health-related quality of life (HRQoL), university, academics, youth

## Abstract

**Objective:**

This study aimed to assess the health-related quality of life (HRQoL) of young academics in Wuhan, China, and its determinants.

**Methods:**

A multistage stratified cluster sampling strategy was employed to recruit study participants (young academics <40 years old) from 12 universities in Wuhan. A total of 301 respondents returned a self-complete questionnaire that contained the EQ-5D-5L. Multivariate linear and Tobit regression models were established to determine the sociodemographic and job predictors of the visual analogue scale (VAS) score and the EQ-5D utility index, respectively.

**Results:**

The study participants reported a mean VAS value of 79.42 (*SD = 10.51*) and a mean EQ-5D utility index of 0.915 (*SD = 0.090*). Anxiety/depression was the most frequently reported problem (65.12%), followed by pain/discomfort (43.52%). Transitioning towards a full professorship in national key universities (*p* < 0.001), lower income (*p* < 0.05) and too much pressure for academic promotion (*p* < 0.001) were significant predictors of lower HRQoL; whereas, maintaining routines in physical activities (*p* < 0.001), sleep (*p* < 0.001) and meals (*p* < 0.001), a good relationship with colleagues and family members (*p* < 0.001), and social activities (*p < 0.01*) were significant predictors of higher HRQoL.

**Conclusion:**

Low HRQoL of young academics in China is evident, as indicated by the 7.08 and 0.049 gap in VAS and utility index, respectively, compared to the general population at the same age. Work and career pressures are associated with the low HRQoL of young academics. The findings of this study highlight the importance of work-life balance in promoting HRQoL of young academics in universities in China.

## Introduction

Academics play a crucial role in scientific advancement, technology innovation, and skilled workforce development. In China, the vast majority of academics are employed in the university sector, of whom more than three quarters are full-time employees (Liu et al., 2015). The 21st century witnessed rapid growth and expansion of the university sector in China, with the number of full-time university academics more than tripled within 20 years. According to the official statistics from the Ministry of Education, China increased its number of university academics from 462,772 in 2000 to 1,740,145 in 2019. Young university academics (≤40 years) account for more than 50% of the academic workforce, exceeding 881,000 in 2019 (Ministry of Education of the People’s Republic of China, 2020a,b). They have become the backbone of the university sector (Chen, 2015), taking 60–80% of teaching loads (Ping, 2015) while maintaining high productivity in research. On average, each young academic in the universities overseen by regional governments published 3.4 peer-reviewed articles per year (Zhe, 2020) indexed by the Science Citation Index, the Social Science Citation Index, the Chinese Science Citation Database, and the Chinese Social Science Citation Index. This figure is even higher (4.21) in the young academics employed by the national key laboratories in universities (Li and Yang, 2019).

Maintaining good health and wellbeing is critical for sustaining the high academic productivity of young university academics (Liu et al., 2015; Ramalho-Pires de Almeida et al., 2019). However, increasing concerns on the health and wellbeing of university academics have been raised in recent years (Chen, 2015; Gao et al., 2019). Reports of sudden deaths of young university academics have become increasingly frequent. In October 2018, two deaths of university academics in their 30th within 1 week attracted great media attention. Overwork (Karoshi) was suspected to be a major contributor to the deaths. This has trigged extensive enquiries into the health and wellbeing of university academics. Indeed, studies have reported poorer health (Gao et al., 2019; Zhang and Xia, 2019) and lower health-related quality of life (HRQoL) of university academics than those of the general populations in China (Tao and Yin, 2006; Ge et al., 2011; Liu et al., 2015; Sanchez et al., 2019). However, there is a paucity in the literature focusing explicitly on the health and wellbeing of young university academics in China (Chen, 2015; Fernandez et al., 2016), despite some evidence about their mental health challenges (Zhu et al., 2003; Tao and Yin, 2006; Zhang, 2014; He et al., 2015; Ping, 2015).

HRQoL is a multi-dimensional concept, which can be defined as “*how well a person functions in their life and his or her perceived wellbeing in physical, mental, and social domains of health*” (Karimi and Brazier, 2016). HRQoL measures health functioning that impacts a person’s daily life, covering personal, family, working, and social domains, which is a more powerful predictor of mortality and mobility than many objective measures of health conditions (DeSalvo et al., 2006). Common instruments assessing HRQoL include the World Health Organization Quality of Life Questionnaire (WHOQOL-100 and WHOQOL-BREF), the EuroQol 5-Dimension (EQ-5D-3L, EQ-5D-5L, and EQ-5D-Y), the Short-Form 36 (SF-36, SF-12, SF-6), the Health Utilities Index (HUI2 and HUI3), the 15-dimensional measure (15D), the Quality of Life Assessment (AQoL-8D), and the Quality of Wellbeing Scale Self-Administered Form (QWB-SA) (Zheng et al., 2021). The WHOQOL-BREF, the SF-6D, and the Total Quality of Work Life (TQWL-42) instruments have been used in assessing the HRQoL of university academics in Thailand (Sriutaisuk, 2014), Malaysia (Naslina et al., 2012; Manaf et al., 2021), Brazil (Sanchez et al., 2019), and Jordan (Almhdawi et al., 2021). In China, the SF-36 is perhaps the most commonly adopted instrument for assessing HRQoL of university employees (Ge et al., 2011; Liu et al., 2015; Yao et al., 2015). Demographic (gender, age), work-related (workload, academic performance), and health (chronic diseases, body weight) factors have been identified as significant predictors of HRQoL of university employees (Ge et al., 2011; Liu and Yi, 2020). However, there is a lack of understanding about how these factors influence the HRQoL of young academics, despite increasing concerns about the health consequences resulting from high academic expectations and pressures on young academics. This study aimed to address the gap in the literature by assessing the HRQoL of young university academics in China. This study also identified personal and job predictors of HRQoL, which is important for better understanding the work environment of young university academics and its association with HRQoL.

## Materials and methods

A cross-sectional survey was conducted on young university academics in Wuhan, China. Ethics approval was obtained from the research committee of the School of Political Science and Public Administration, Wuhan University (Project number 201910486033). The study was executed in accordance with the 1964 Helsinki declaration and its later amendments.

### Study setting

The study was conducted in Wuhan, the capital of Hubei province in central China. Wuhan has 83 tertiary education institutions, including 18 universities. They employ over 93,400 workers, among whom about 59,600 (63.8%) were full-time academics. Tertiary student enrolments reached 1,126,197 in 2019 in Wuhan, ranking on top of all cities in China.

### Sampling and data collection

A multistage stratified cluster sampling strategy was adopted to recruit study participants from the universities in Wuhan. Eligible participants were full-time academics who were younger than 40 years (born after 1 January 1979). The 18 universities in Wuhan were categorised into three tiers in line with the classification of the Ministry of Education in China: two in the top tier funded by the national “985 project” (initiated in 1999); five in the middle tier tagged as key universities in the national “211 project” (initiated in 1995); and 11 in the low tier overseen by the provincial government. At the first stage, all of the top (2) and middle (5) tier universities were selected, along with five universities in the low tier randomly selected. At the second stage, the academic staff profiles published on the webpages of the participating universities were examined to identify eligible participants: excluding those who claimed an age of over 40 years. This resulted in a total of 3,012 email contacts. An email invitation was then sent to those identified on 1 May 2019. Eventually, 323 (10.7%) questionnaires were returned through email with implied informed consent and 301 were included for the final data analysis after further excluding 22 questionnaires that contained missing data about the key measurement (EQ-5D-5L) or/and reported an age older than 40 years.

### Measurement

The survey contained three sections: sociodemographic characteristics, the EQ-5D-5L instrument, and life and work conditions.

#### Dependent variable

The primary interest of this study was HRQoL of the study participants. Several instruments were available in China assessing HRQoL. These included the EQ-5D, the SF-36 and its shorter versions (SF-12 and SF-6D), the HUI, and the WHOQOL-BREF (Organization, W. H., 2004; Németh, 2006; Tan et al., 2013). The EQ-5D is perhaps the most widely used one for its easiness to be administered, scored, and interpreted. It has more than 170 language versions, including in Chinese, and has been widely used for assessing HRQoL in various populations under various settings (Lou et al., 2015; Xu et al., 2016; Feng et al., 2018; Huang et al., 2018; Zhou et al., 2018; Zeng et al., 2021).

The Chinese version of EQ-5D instruments has demonstrated good reliability and validity (Wang et al., 2012; Fang et al., 2016), including the EQ-5D-5L (Xie et al., 2022). This study adopted the Chinese version of EQ-5D-5L (Luo et al., 2013). A value set for the EQ-5D-5L based on Chinese population preferences was created that allows the calculation of health utility (Luo et al., 2017). Recently, Yang and colleagues established the EQ-5D-5L population norms for urban residents by various age groups (Yang et al., 2018).

Respondents of this study were asked to report health problems measured by the EQ-5D-5L in relation to mobility (MO), self-care (SC), usual activities (UA), pain/discomfort (PD), and anxiety/depression (AD), each being rated on a five-level scale ranging from “no problem” to “extreme difficulty” (Huang et al., 2017). The combination of the reported problems was converted into a utility index between 0 (indicating “death”) and 1 (indicating “full health”) using the population preference-based value set in China (Luo et al., 2017). In addition, the study participants were asked to rate their overall health along a Visual Analogue Scale (VAS) ranging from 0 (worst possible) to 100 (best possible) (Yao et al., 2019).

#### Independent variables

Selection of the independent variables that predict HRQoL was guided by the social ecology theory (Fan et al., 2012; Golden et al., 2015) and the job demands-control theory (Karasek, 1979; Zhang et al., 2017). The social ecology theory classifies health determinants into three levels: (1) individual-sociodemographic characteristics and lifestyle choice; (2) family and social networks; and (3) organisational and work environments (Fan et al., 2012). Young university academics are establishing their professional career and family nests simultaneously, which may reinforce each other in building accumulative pressures and stress. The job demands-control theory provides a framework interpreting how organisational and work conditions affect individual workers. It was first proposed by Karasek in 1979 in the context of increasingly prominent employee health problems caused by workload (Karasek 1979). In this theory, job demands represent the source of work pressure. Individuals cope with job demands through various control measures.

The sociodemographic data collected in this study included gender (male and female), age (≤30, 31–35, and 36–40 years), and educational attainment. Maintenance of routines in physical activities (yes, no), sleep (yes, no), and meals (yes, no) over the past month was measured to reflect lifestyle choice because they were identified in previous studies as common problems experienced by young university academics (Zhao et al., 2012).

Family and social networks were assessed by personal relationships with colleagues, family functioning, and participation in social activities. Respondents were asked to rate their perceptions on a five-point Likert scale ranging from “1 = totally disagree” to “5 = totally agree,” with a higher score indicating higher availability of family and social networks. The rating scales were collapsed into three categories (disagree, neutral, agree) for data analyses.

Organisational and work conditions were measured by job title (lecturer, associate professor, professor), contract (permanent, non-permanent), and annual salary after tax (≤100,000, 100,001–150,000, and > 150,000 Chinese Yuan). These conditions are associated with job demands. Respondents were asked to report felt pressure in teaching, research, and promotion on a five-point Likert scale (ranging from “1 = totally disagree” to “5 = totally agree”) in line with previous studies (Luo et al., 2017), with a higher score indicating higher pressure. The rating scales were collapsed into three categories (disagree, neutral, agree) for data analyses.

Significant variations in teaching and research requirements exist across the three tiers of universities in China. The top two tiers of universities attracted the vast majority of research funding and produced the most research outputs. The research benchmark for academics in the higher tiers of universities is usually higher compared with those working in the lower tiers of universities.

### Statistical analysis

The percentage of respondents reporting problems on each health dimension and the mean utility index and VAS scores (SD: Standard Deviations) of the EQ-5D-5L were calculated, and compared with the population (20–39 years old) norms (Yang et al., 2018). Chi-square tests were performed to test group differences in the frequency of reported health problems. Student *t* tests or one-way analysis of variance (ANOVA) were performed to test group differences of the utility index and VAS scores.

Multivariate Tobit and linear regression models were established to determine the predictors of the EQ-5D utility index and VAS scores, respectively. A Tobit model was applied for the utility index because its score was censored at 1.00 (Zhang et al., 2016). Given that the data were not always normally distributed, the modelling was re-run using Box-Cox transformation (Supplementary File) to verify the robustness of the results (Bicego and Baldo, 2016; Guirant et al., 2018; Rand et al., 2020). The regression models contained two levels of independent variables: the individual characteristics of respondents were nested in the cluster of universities. Given the relatively small intra-class correlations (0.03–0.05), we present the single-level (individual measurements only) regression results. The results of the two-level regression models with a random intercept effect (Supplementary File) are largely consistent with the single-level models. All of the independent variables were coded as dummy variables in the regression models. The variance–covariance matrix and standard errors were estimated using the robust method.

All statistical analyses were carried out using STATA IC 16.0 for Windows.

## Results

### Characteristics of respondents

The respondents had a mean age of 34.79 years (*SD =* 3.54) and 70.43% were male. Early career academics (lecturers) accounted for more than half of the respondents. More than 70% entered a permanent contract with their university. The salary level was high, with 83.39% earning an annual salary over 100,000 Chinese Yuan after tax, compared with an average income of 51,706 Chinese Yuan in Wuhan (Table 1).

**Table 1:** Characteristics of respondents (*n* = 301).

**Characteristics**		**N**	**%**
**Personal characteristics**			
***Gender***			
	Male	212	70.43
	Female	89	29.57
***Age (Years)***			
	21–30	34	11.30
	31–35	126	41.86
	36–40	141	46.84
***Annual salary after-tax (Chinese Yuan)***			
	≤100,000	50	16.61
	100,000–150,000	180	59.80
	>150,000	71	23.59
**Health-related behaviors**			
***Regular physical activities***			
	Yes	99	32.89
	No	202	67.11
***Regular sleeping***			
	Yes	180	59.80
	No	121	40.20
***Regular meals***			
	Yes	254	84.39
	No	47	15.61
**Family and social networks**			
***Actively participate in social activities***			
	Disagree	31	10.30
	Neutral	100	33.22
	Agree	170	56.48
***Get along well with colleagues***			
	Disagree	6	1.99
	Neutral	43	14.29
	Agree	252	83.72
***Harmonious family relationship***			
	Disagree	743.52	2.33
	Neutral	25	8.31
	Agree	269	89.37
**Organisational and working condition**			
***University***			
	Top tier (985 project)	35	11.63
	Mid tier (211 project)	156	51.83
	Low tier (Provincial)	110	36.54
***Job title***			
	Lecturer or below	158	52.49
	Associate professor	119	39.53
	Full professor	24	7.97
***Employment contract***			
	Permanent	211	70.10
	Non-permanent	90	29.90
***Too much pressure on teaching***			
	Disagree	35	11.63
	Neutral	79	26.25
	Agree	187	62.13
***Too much pressure on research***			
	Disagree	10	3.32
	Neutral	30	9.97
	Agree	261	86.71
***Too much pressure on academic promotion***			
	Disagree	19	6.31
	Neutral	34	11.30
	Agree	248	82.39
**Total**		301	100.00

Over the past month, two thirds (67.11%) of respondents did not engage in regular physical exercises; 40.20% lacked regular sleeping; 15.61% missed regular meals; and 43.52% did not participate in any social activities. The vast majority (83.72%) reported good relationships with colleagues and family members. High levels of pressure to meet performance expectations were felt, with 62.13% reporting “too much pressure” on teaching, 86.71% reporting “too much pressure” on research, and 82.39% reporting “too much pressure” for academic promotion (Table 1).

### HRQoL of respondents

**Figure 1:**
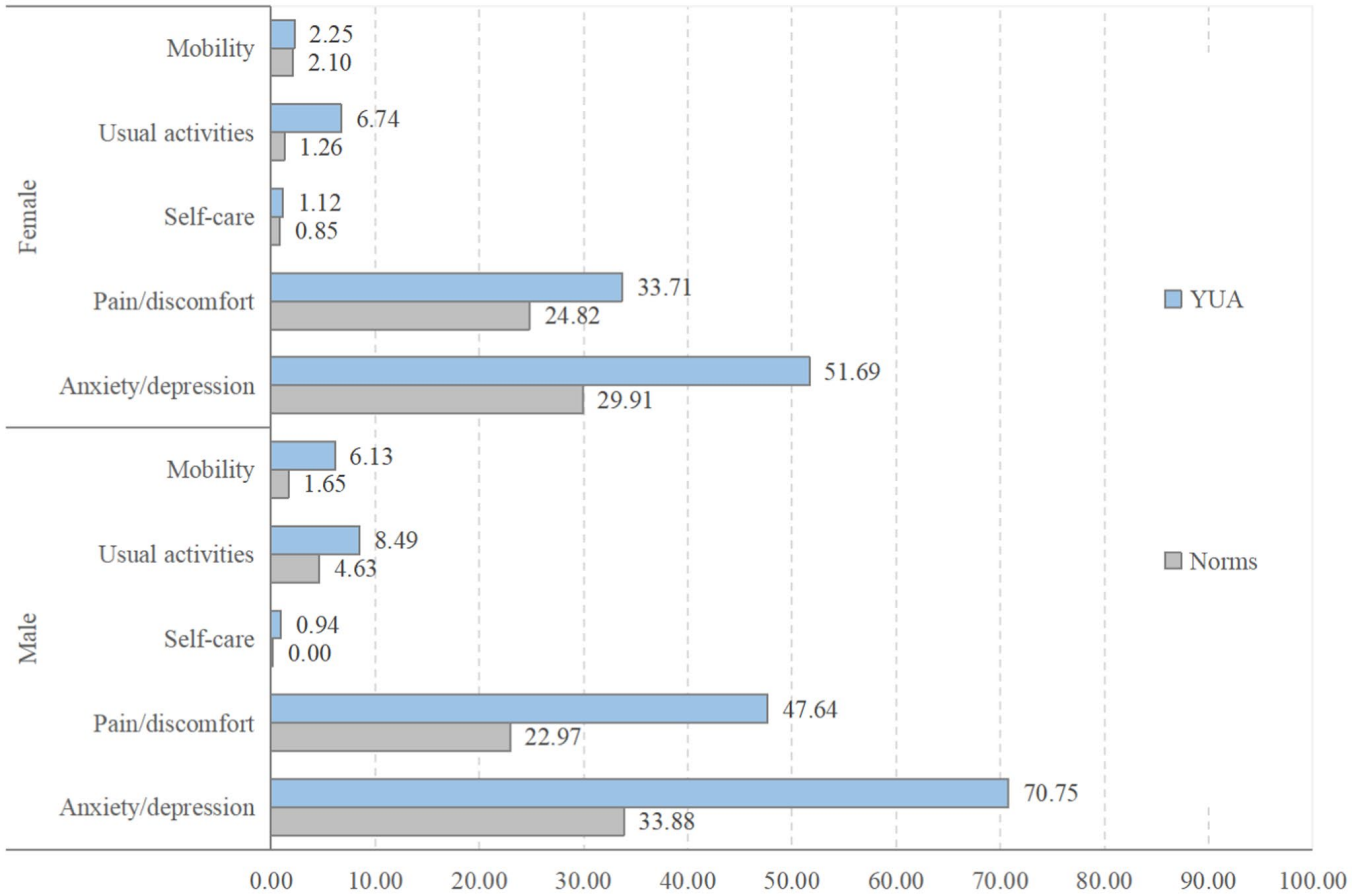
Percentages of respondents reporting health problems in comparison with population norms by gender. YUA – Young University Academics involved in this study. Data of population norms: Yang et al. (2018).

Overall, 25.58% of respondents did not report any problem across the five health dimensions of the EQ-5D-5L, compared with 62.93% in the general population at the same age range (*p* < 0.001). Higher percentages of problems were reported in all of the five health dimensions in comparison with the general population at the same age range: 65.12% vs. 31.92% in anxiety/depression (*p* < 0.001); 43.25% vs. 23.88% in pain/discomfort (*p* < 0.001); 7.97% vs. 2.97% in usual activities (*p < 0.01*), 4.98% vs. 1.87% in mobility (*p* < 0.05), and 1.00% vs. 0.42% in self-care (*p = 0.331*). Such gaps existed in both genders (Figure 1).

On average, the respondents had a VAS score 79.42 (*SD = 10.51*) and a utility index score 0.915 (*SD = 0.090*), 7.08 (*p* < 0.001), and 0.049 lower (*p* < 0.001) than that of the general young population, respectively. Such gaps existed in both genders, albeit more profound in the men compared with the women (8.11 vs. 4.19 for VAS; 0.057 vs. 0.024 for utility index) (Figure 1).

### Factors associated with HRQoL

Female respondents and those who maintained routines in regular physical activities, sleep and meals, and participated in social activities were less likely to report health problems, resulting in higher VAS and utility index scores (*p* < 0.05). Higher availability of family and social networks was associated with higher VAS and utility index scores (*p* < 0.05), despite a lack of statistical differences in the percentage of reported health problems. Several indicators of work conditions were found to be associated with HRQoL. The respondents from the top tier (985 project) universities had significantly lower scores in VAS (*p* < 0.001) and utility index (*p* < 0.05). Permanent employment, transitioning to full professorship, and high teaching pressure were associated with higher reported problems and/or lower EQ-5D scores (*p* < 0.05) (Table 2).

**Table 2:** Percentage of respondents reporting health problems and VAS and utility index scores.

**Characteristics of respondents**		**Percentage (%) of respondents reporting problems**					**Mean (95% CI) score of VAS**	**Mean (95% CI) score of utility index**
		**Mobility**	**Self-care**	**Usual activities**	**Pain/ discomfort**	**Anxiety/ depression**		
***Gender***								
	Male	6.13%	0.94%	8.49%	47.64%	70.75%	78.07 (76.60, 79.54)	0.905 (0.891, 0.918)
	Female	2.25%	1.12%	6.74%	33.71%	51.69%	82.64 (80.76, 84.52)	0.941 (0.927, 0.934)
*p*		*0.158*	*0.886*	*0.609*	0.026**	0.002***	<0.001****	0.001***
***Age groups (Years)***								
	21–30	2.94%	2.94%	2.94%	29.41%	58.82%	80.76 (77.52, 84.01)	0.935 (0.912, 0.958)
	31–35	4.76%	0.79%	9.52%	41.27%	66.67%	79.83 (77.79, 81.86)	0.914 (0.898, 0.930)
	36–40	5.67%	0.71%	7.80%	48.94%	65.25%	78.74 (77.10, 80.37)	0.911 (0.896, 0.927)
*p*		*0.797*	*0.479*	*0.451*	0.096*	*0.695*	*0.513*	*0.391*
***Annual salary after-tax (Chinese Yuan)***								
	≤100,000	6.00%	2.00%	12.00%	40.00%	60.00%	80.30 (77.00, 83.60)	0.911 (0.880, 0.943)
	100,000–150,000	3.89%	0.56%	6.11%	45.56%	67.22%	79.56 (78.06, 81.07)	0.913 (0.900, 0.926)
	>150,000	7.04%	1.41%	9.86%	40.85%	63.38%	78.45 (76.98, 80.92)	0.923 (0.904, 0.942)
*p*		*0.549*	*0.610*	*0.317*	*0.683*	*0.626*	*0.612*	*0.702*
***Regular physical activities***								
	Yes	3.03%	0.00%	3.03%	39.39%	55.56%	81.98 (80.14, 83.82)	0.936 (0.925, 0.948)
	No	5.94%	1.49%	10.40%	45.54%	69.80%	78.17 (76.66, 79.68)	0.905 (0.891, 0.918)
*p*		*0.276*	*0.223*	0.027**	*0.312*	0.015**	0.003***	0.004***
***Regular sleeping***								
	Yes	5.00%	1.11%	4.44%	36.67%	58.33%	81.31 (79.78, 82.83)	0.930 (0.918, 0.941)
	No	4.96%	0.83%	13.22%	53.72%	75.21%	76.62 (74.80, 78.44)	0.894 (0.875, 0.913)
*p*		*0.987*	*0.807*	0.006***	0.003***	0.003***	<0.001****	<0.001****
***Regular meals***								
	Yes	4.72%	1.18%	7.48%	42.52%	62.20%	80.69 (79.42, 82.96)	0.920 (0.910, 0.931)
	No	6.38%	0.00%	10.64%	48.94%	80.85%	72.55 (69.87, 75.24)	0.888 (0.856, 0.922)
*p*		*0.631*	*0.454*	*0.463*	*0.415*	0.014**	<0.001****	0.029**
***Actively participate social activities***								
	Disagree	9.68%	3.23%	6.45%	58.06%	74.19%	72.61 (68.59, 76.64)	0.867 (0.824, 0.911)
	Neutral	5.00%	0.00%	8.00%	51.00%	79.00%	77.24 (75.27, 79.21)	0.898 (0.878, 0.914)
	Agree	4.12%	1.18%	8.24%	36.47%	55.29%	81.95 (80.44, 83.45)	0.934 (0.923, 0.945)
*p*		*0.425*	*0.269*	*0.945*	0.015**	<0.001****	<0.001****	<0.001****
***Get along well with colleagues***								
	Disagree	16.67%	0.00%	16.67%	66.67%	83.33%	77.17 (63.16, 91.17)	0.852 (0.737, 0.968)
	Neutral	6.98%	2.33%	9.30%	41.86%	79.07%	75.77 (72.23, 79.31)	0.886 (0.853, 0.919)
	Agree	4.37%	0.79%	7.54%	43.25%	62.30%	80.10 (78.84, 81.36)	0.922 (0.911, 0.932)
*p*		*0.318*	*0.626*	*0.675*	*0.506*	0.066*	0.038**	0.012**
***Harmonious family relationship***								
	Disagree	0.00%	0.00%	14.29%	71.43%	85.71%	64.29 (52.22, 76.35)	0.837 (0.748, 0.925)
	Neutral	12.00%	0.00%	8.00%	52.00%	72.00%	74.72 (69.62, 79.82)	0.886 (0.840, 0.931)
	Agree	4.46%	1.12%	7.81%	42.01%	63.94%	80.25 (79.07, 81.44)	0.920 (0.910, 0.930)
*p*		*0.210*	*0.835*	*0.823*	*0.202*	*0.369*	<0.001****	0.012**
***University***								
	Top tier (985 project)	11.43%	0.00%	14.29%	57.14%	77.14%	73.46 (69.76, 77.16)	0.882 (0.846, 0.918)
	Mid tier (211 project)	4.49%	1.28%	7.69%	41.67%	60.26%	81.00 (79.36, 82.64)	0.915 (0.900, 0.931)
	Low tier (Provincial)	3.64%	0.91%	6.36%	41.82%	68.18%	79.08 (77.19, 81.97)	0.926 (0.913, 0.938)
*p*		*0.168*	*0.783*	*0.316*	*0.224*	*0.116*	<0.001****	0.044**
***Job title***								
	Lecturer or below	4.43%	0.63%	6.96%	35.44%	59.49%	80.82 (79.05, 82.58)	0.928 (0.915, 0.940)
	Associate professor	6.72%	1.68%	10.08%	56.30%	74.79%	77.81 (76.08, 79.54)	0.892 (0.874, 0.911)
	Full professor	37.50%	0.00%	4.17%	33.33%	54.17%	78.25 (74.33, 82.17)	0.947 (0.924, 0.969)
*p*		*0.346*	*0.601*	*0.492*	0.001***	0.015**	*0.052*	0.001***
***Employment contract***								
	Permanent	6.16%	1.42%	8.06%	45.50%	70.62%	79.28 (77.93, 80.63)	0.908 (0.895, 0.921)
	Non-permanent	2.22%	0.00%	8.89%	38.89%	52.22%	79.76 (77.30, 82.21)	0.932 (0.917, 0.947)
*p*		*0.150*	*0.256*	*0.935*	*0.290*	0.002***	*0.720*	0.036**
***Too much pressure on teaching***								
	Disagree	5.71%	0.00%	2.86%	37.14%	51.43%	82.80 (79.29, 86.31)	0.933 (0.904, 0.962)
	Neutral	1.68%	1.12%	2.23%	18.44%	26.26%	80.63 (78.40, 82.87)	0.924 (0.905, 0.943)
	Agree	5.35%	0.53%	10.16%	45.45%	70.05%	78.28 (76.74, 79.81)	0.908 (0.895, 0.922)
*p*		*0.849*	*0.267*	*0.185*	*0.618*	0.05**	0.0315**	*0.194*
***Too much pressure on research***								
	Disagree	0.00%	0.00%	0.00%	40.00%	50.00%	82.60 (75.82, 89.38)	0.921 (0.844, 0.997)
	Neutral	3.33%	0.00%	3.33%	26.67%	53.33%	82.43 (78.20, 86.66)	0.947 (0.922, 0.973)
	Agree	5.36%	1.15%	8.05%	45.59%	67.05%	78.95 (77.69, 80.22)	0.911 (0.900, 0.922)
*p*		*0.678*	*0.793*	*0.368*	*0.137*	*0.195*	*0.143*	*0.112*
***Too much pressure on academic promotion***								
	Disagree	5.26%	0.00%	5.26%	42.11%	52.63%	84.53 (80.45, 88.60)	0.920 (0.871, 0.968)
	Neutral	8.82%	0.00%	8.82%	29.41%	64.71%	78.91 (74.81, 83.01)	0.920 (0.878, 0.961)
	Agree	4.42%	1.20%	8.03%	45.38%	65.86%	79.10 (77.80, 80.40)	0.914 (0.904, 0.925)
*p*		*0.544*	*0.723*	*0.893*	*0.203*	*0.492*	*0.091*	*0.925*
**Total**		4.98%	1.00%	7.97%	43.52%	65.12%	79.42 (78.23, 80.61)	0.915 (0.905, 0.935)

*p < 0.10; **p < 0.05; ***p < 0.01; ****p < 0.001.

The multivariate regression models showed that regular physical exercises, social activities, and availability of family support were significant predictors of both higher VAS and higher utility index scores (*p* < 0.05), while transitioning towards full professorship and working in top-tier (985 project) universities were significant predictors of both lower VAS and lower utility index scores (*p* < 0.05), after adjustment for variations in other variables. Lower salary and a lack of regular sleep were significant predictors of lower utility index (*p* < 0.05), but not for VAS. Regular meals, lower pressures on teaching and for academic promotion, non-permanent contracts, and collegial support were significant predictors of higher VAS scores (*p* < 0.05), but not for utility index (Table 3). The Box-cox transformation generated consistent results, except for the lack of significant association between teaching and VAS scores (*p* < 0.05) (Supplementary File).

**Table 3:** Predictors of VAS and utility index scores: results of single-level linear and Tobit regression models.

**Variables**		**Linear regression on VAS**		**Tobit regression on utility index**	
		***β***	**95% CI**	***β***	**95% CI**
***Gender (Reference: Male)***					
	Female	1.13	(−1.22, 3.48)	0.02	(−0.01, 0.04)
***Age group (years) (Reference: 21–30)***					
	31–35	0.54	(−2.75, 3.82)	−0.01	(−0.05, 0.03)
	36–40	0.39	(−3.12, 3.91)	0.00	(−0.04, 0.04)
***Annual salary after-tax (Chinese Yuan) (Reference: ≤100,000)***					
	100,000–150,000	0.42	(−2.86, 3.69)	0.02	(−0.02, 0.05)
	>150,000	1.19	(−3.13, 5.51)	0.05**	**(>0.00, 0.10)**
***Regular physical activities (Reference: Yes)***					
	No	−2.33**	**(−4.40, −0.27)**	−0.03**	**(−0.06, −0.01)**
***Regular sleeping (Reference: Yes)***					
	No	−1.02	(−3.34, 1.29)	−0.03**	**(−0.05, −0.01)**
***Regular meals (Reference: Yes)***					
	No	−6.20****	**(−9.20, −3.19)**	−0.01	(−0.05, 0.03)
***Actively participate in social activities (reference: disagree)***					
	Neutral	5.59***	**(1.77, 9.40)**	0.03	(−0.02, 0.07)
	Agree	8.29****	**(4.44, 12.14)**	0.06**	**(0.01, 0.10)**
***Get along well with colleagues (Reference: Disagree)***					
	Neutral	12.19***	**(5.28, 19.10)**	−0.02	(−0.12, 0.08)
	Agree	12.09****	**(5.59, 18.58)**	0.01	(−0.09, 0.10)
***Harmonious family relationship (reference: disagree)***					
	Neutral	17.21****	**(9.23, 25.19)**	0.08**	**(0.01, 0.15)**
	Agree	21.10****	**(13.90, 28.30)**	0.09**	**(0.02, 0.16)**
***Type of university (Reference: Top tier (985 project))***					
	Mid tier (211 project)	6.18***	**(2.12, 10.25)**	0.06***	**(0.02, 0.10)**
	Low tier (Provincial)	3.43	(−1.00, 7.85)	0.07***	**(0.03, 0.12)**
***Job title (Reference: Lecturer or below)***					
	Associate professor	−2.87**	**(−5.37, −0.36)**	−0.04**	**(−0.06, −0.01)**
	Full professor	−3.72	(−8.99, 1.54)	0.02	(−0.03, 0.07)
***Employment contract (Reference: Permanent)***					
	Non-permanent	0.57	(−2.24, 3.39)	0.04***	**(0.01, 0.07)**
***Too much pressure on teaching (Reference: Disagree)***					
	Neutral	0.38	(−3.04, 3.78)	−0.01	(−0.05, 0.03)
	Agree	−2.86*	**(−6.20, 0.49)**	−0.03	(−0.07, 0.02)
***Too much pressure on research (Reference: Disagree)***					
	Neutral	−1.43	(−7.94, 5.07)	0.00	(−0.10, 0.09)
	Agree	−2.53	(−8.97, 3.91)	−0.06	(−0.15, 0.03)
***Too much pressure on academic promotion (Reference: Disagree)***					
	Neutral	−5.03	(−10.37, 0.30)	0.02	(−0.05, 0.09)
	Agree	−4.60*	**(−9.24, 0.03)**	0.03	(−0.03, 0.10)

*p < 0.1; **p < 0.05; ***p < 0.01; ****p < 0.001.

## Discussion

Lower HRQoL of young university academics was found in this study, as indicated by the 7.08 and 0.049 gaps in VAS and utility index, respectively, compared to the general population at the same age. Our study participants reported more health problems in all of the five dimensions measured by the EQ-5D-5L. These results are consistent with the findings of previous studies (Ge et al., 2011; Yang et al., 2018). Anxiety/depression and pain/discomfort appear to be common complaints of university academics (Ge et al., 2011; Liu et al., 2015; Ping, 2015; Fernandez et al., 2016; Sanchez et al., 2019). Ergonomic factors (sitting, elevated shoulder positions, and standing) are often blamed for the pain/discomfort (Constantino Coledam et al., 2019). Young academics in China undertake more than 70% of university course loads (Zhe, 2020). Most of them also experience intense mental exertion in creative research tasks. Adding to the work pressure is the tedious process of administrative tasks and meetings (Barnett et al., 2019; Sanchez et al., 2019). The conflict between high work demands and high family duties can further exacerbate the emotional and mental exhaustion of young academics, making them feel vulnerable, irritable, and anxious (Reevy and Deason, 2014; Meng and Wang, 2018; Sanchez et al., 2019; Xu, 2019).

We found that job performance pressure relating to teaching and academic promotion is a significant predictor of low HRQoL of young academics. Similar findings have been reported in the studies conducted elsewhere in China (Li and Kou, 2018; Liu and Yi, 2020) and Brazil (Sanchez et al., 2019). In recent years, universities in China have gone through various ranking systems domestically and internationally. The rapid rising of Chinese universities in the ranking systems is accompanied by unprecedented pressure on the performance of university academics. Previous studies showed that it is not uncommon for young university academics to stay up late and suffer from sleep deprivation under high work stress (Macaluso et al., 2015; Zhang et al., 2016; Huang et al., 2017; Sanchez et al., 2019). In our study, lower HRQoL was found to be associated with a lack of regular sleep, meals, and physical activities. “Allostatic load” has often been used to explain the physiological and psychological attrition that accumulates in the human body during prolonged stressful conditions (McEwen and Stellar, 1993). Constant exposure to endogenous and exogenous stimuli can lead to chronic stress changes of the human body as a result of responses from the nervous and endocrine systems. The consequences individuals experience range from stress, anxiety, headaches, insomnia, and impaired functioning of daily lives to cardiovascular disease, depression, diabetes, and even death (Bellingrath et al., 2009; Ganster and Rosen, 2013; Bo et al., 2021).

Young university academics transitioning to a full professorship reported lower HRQoL than their junior and senior counterparts in our study. This finding is supported by a study of young academics in 25 universities in Zhejiang province (He et al., 2015) and a survey of university academics of all ages in 64 colleges in Liaoning province (Ge et al., 2011). With the rising ranking and performance requirements, universities in China have been engaged in intense competition to attract talented young academics through high salary packages. However, expectations on the academic performance of the new hires are high. Transitioning to full professorship is consequently a stressful journey. Compared to the older generations, the bar for promotion to professor is usually set much higher. Young academics with a title of associate professor not only need to excel in research performance but also need to excel in teaching. It is compulsory for them to teach undergraduate courses with large student enrolments and take duties of student administration. This can often lead to increased worry about research performance (Meng and Wang, 2018). Associate professors often find their research time being crowded out by teaching commitments and have to extend work time and cut back breaks and vacations to maintain competitiveness. Positions available for promotion are always limited in Chinese universities. Meeting or exceeding the promotion bar does not necessarily guarantee a chance of promotion (Hvistendahl, 2013; Huang, 2018). Young academics have to work extraordinarily hard to become an exemplar. Although the “culture of overwork” is common across the entire academic community in China (Barnett et al., 2019), Qiu and colleagues found that associate professors have worse mental health than professors (Qiu et al., 2007).

We found that young academics working in the top tier universities reported worse HRQoL than their colleagues in the lower tier universities. Higher performance benchmarking can be an additional source of work stress in top universities (Liu et al., 2015). The “985 project” aims to build “first-class universities” and “first-class disciplines” in the world. Therefore, global benchmarking applies. While the vast majority of research funding goes to top tier universities, young academics working at top universities are facing higher expectations relative to those in other universities on grant success and the impacts of research outcomes (Chen et al., 2021). Yan reported that 3,650 academics in the 35 top universities (985 project) work 59 h per week on average (Yan, 2018).

Permanent employment does not improve the HRQoL of young university academics, according to the findings of this study. This is contradictory to the job security theory (Wagenaar et al., 2012). In the Chinese context, permanent academic employees are still facing a high level of uncertainty in career success. There exists intense competition among colleagues for limited promotion opportunities, which can jeopardise collegial support and work conditions (Zhang, 2014). Those who fail to progress through academic promotion would virtually be demoted. In recent years, a tenure track system (also named as “Up-or-Out”) has increasingly been endorsed by the university sector, in particular those in the top tier. It sets up a rigorous timeline for young academics to progress through the academic career system. Candidates are ranked in orders (Musselin, 2005). Those who are ranked at the bottom may not have their contracts renewed (Wang and Jones, 2021), leading to a feeling of job insecurity (Tian and Lu, 2017; Liu et al., 2021). Overwork has thus become a self-adaptive norm (Si, 2022).

The work requirement-control theory proposes some mechanisms for improving HRQoL (Zhang et al., 2017). For example, material and social support can help mitigate some of the health risks imposed by high work stress. Social support has been proved to reduce the psychological and physiological consequences of stress and may enhance immune function (Heinze et al., 2015; Harandi et al., 2017; Xu, 2019). Indeed, our study and several other studies (Zhou et al., 2018; Almhdawi et al., 2021) show that higher income is a predictor of higher HRQoL. Higher income reduces financial pressure and enables high standards of living, which can contribute to better health. Empirical evidence shows that good relationships with colleagues and a well-functioning family can also offer support to young university academics in dealing with the work stress that impacts their HRQoL (Ge et al., 2011; He et al., 2015; Liu et al., 2015). Our study provides additional evidence to support such an argument. We also found that participation in social activities is positively associated with HRQoL.

Findings of this study have some policy implications. Ensuring the health and wellbeing of young academics is fundamental for the sustainable development of universities in China. This requires a good design of career pathway and proper work-life balance. It is important to note that academic overwork in universities is not unique to China. In the UK, for example, 42% of university academics believed that they have to devote private time to work (Kinman and Jones, 2004), and only those who are able to conduct research in their private time are likely to progress professionally (Barrett and Barrett, 2010).

The physical and mental wellbeing of young academics is not just an issue of occupational health and safety, it is strategically important for the sustainable academic development and innovations in universities. Academic productivity can and should be maintained through a culture and environment that puts employee well-being at the centre. This requires flexible work arrangements that address work-life balance, individualised career design, and strong professional and social support (Zhang, 2014; Chen, 2015; He et al., 2015; Ping, 2015; Fernandez et al., 2016). Individuals, families, universities, and the broad society all play a role in the process (Li and Zeng, 2010). Recently, the Chinese government has started to reform its academic performance assessment and professional promotion system in order to nurture a supportive environment for university academics that encourages academic innovations and long-term advancement of the university sector.

This study has several limitations. Firstly the sample size of this study is relatively small. Secondly, study participants were recruited from one city in China and were restricted to the full-time employees. Attempts to generalise the findings should be cautious. Future studies should consider expansion of the scope of study settings and study participants.

## Conclusion

Low HRQoL of young university academics is evident in China. Work and career pressures are associated with the low HRQoL of young academics. The findings of this study highlight the importance of work-life balance in promoting HRQoL of young academics in universities in China. Building a strong social network may mitigate some of the health risks resulting from high work pressures, but a fundamental solution requires a systems approach. Although a high level of remunerations can help attract talented young academics, it may not be enough to retain them. University managers need to take additional measures to maintain a sustainable academic workforce. This includes, but is not limited to, a strong caring culture and supportive measures such as those relating to health-promoting universities (Liu et al., 2015; Fernandez et al., 2016; Shah et al., 2020).

## Data availability statement

The original contributions presented in the study are included in the article/Supplementary material, further inquiries can be directed to the corresponding authors.

## Ethics statement

The studies involving human participants were reviewed and approved by the research committee of the School of Political Science and Public Administration, Wuhan University (Project number 201910486033). The patients/participants provided their written informed consent to participate in this study.

## Author contributions

QY contributed to the study design, data analyses, and drafting of the manuscript. FY, HL, and KT contributed to the data analyses, data interpretation, and drafting of the manuscript. CL contributed to the interpretation of results and writing of the manuscript. All authors have read and approved the final version of the manuscript.

## Funding

The study was funded by the National Natural Science Foundation of China (72174149 and 71603188), the Humanity and Social Science Foundation from the Ministry of Education of China (21YJAZH102), and Key Research Institute Project of Humanity and Social Science of the Ministry of Education of China (1203–413100050).

## Conflict of interest

The authors declare that the research was conducted in the absence of any commercial or financial relationships that could be construed as a potential conflict of interest.

## Publisher’s note

All claims expressed in this article are solely those of the authors and do not necessarily represent those of their affiliated organizations, or those of the publisher, the editors and the reviewers. Any product that may be evaluated in this article, or claim that may be made by its manufacturer, is not guaranteed or endorsed by the publisher.
