# Supplementary material for: Health-related quality of life of young academics: A cross-sectional survey of universities in Wuhan, China
Source: Front Psychol. 2022 Nov 10;13:996219. doi: 10.3389/fpsyg.2022.996219 (PMC9686858; doi:10.3389/fpsyg.2022.996219)
Supplement: Supplementary file 1 [file Data_Sheet_1.docx]

| **Table S1. Predictors of VAS and utility index scores transformed by Box-Cox: results of single-level linear and Tobit regression models** | | | | | |
| --- | --- | --- | --- | --- | --- |
| **Variables** | | **Linear regression on VAS transformed by Box-Cox**  **(θ=2.5)** | | **Tobit regression on utility index transformed by Box-Cox**  **(θ=6.0)** | |
|  |  | **β** | **95% CI** | **β** | **95% CI** |
| **Gender (Reference: Male)** | |  |  |  |  |
|  | Female | 807.13 | (-850.64, 2464.90) | 0.01 | (<0.01, 0.02) |
| **Age group (years) (Reference: 21-30)** | |  |  |  |  |
|  | 31-35 | 704.14 | (-1606.91, 3015.18) | <0.01 | (-0.02, 0.01) |
|  | 36-40 | 449.58 | (-2027.44, 2926.59) | <0.01 | (-0.02, 0.02) |
| **Annual salary after-tax (Chinese Yuan) (Reference: ≤100000)** | |  |  |  |  |
|  | 100000-150000 | 91.51 | (-2150.39, 2333.41) | <0.01 | (-0.01, 0.02) |
|  | >150000 | 707.48 | (-2196.41, 3611.38) | **0.02**** | **(<0.01, 0.03)** |
| **Regular physical activities (Reference: Yes)** | |  |  |  |  |
|  | No | **-1448.30*** | **(-2895.38, -1.22)** | **-0.01**** | **(-0.02, <0.01)** |
| **Regular sleeping (Reference: Yes)** | |  |  |  |  |
|  | No | -805.79 | (-2403.60, 792.03) | **-0.01**** | **(-0.02, <0.01)** |
| **Regular meals (Reference: Yes)** | |  |  |  |  |
|  | No | **-4248.70****** | **(-6127.33, -2370.07)** | <0.01 | (-0.02, 0.01) |
| **Actively participate in social activities (reference: disagree)** | |  |  |  |  |
|  | Neutral | **3617.50***** | **(1199.32, 6035.67)** | 0.01 | (-0.01, 0.03) |
|  | Agree | **5689.76****** | **(3197.25, 8182.27)** | **0.02**** | **(<0.01, 0.04)** |
| **Get along well with colleagues (Reference: Disagree)** | |  |  |  |  |
|  | Neutral | **7805.18****** | **(3513.20, 12097.16)** | 0.01 | (-0.03, 0.05) |
|  | Agree | **7756.16****** | **(3683.08, 11829.25)** | <0.01 | (-0.04, 0.04) |
| **Harmonious family relationship (reference: disagree)** | |  |  |  |  |
|  | Neutral | **10269.72****** | **(5704.40, 14835.03)** | **0.03**** | **(<0.01, 0.07)** |
|  | Agree | **12570.71****** | **(8506.02, 16635.40)** | **0.04***** | **(<0.01, 0.07)** |
| **Type of university (Reference: Top tier (985 project))** | |  |  |  |  |
|  | Mid tier (211 project) | **4190.08***** | **(1619.96, 6760.21)** | **0.02***** | **(<0.01, 0.04)** |
|  | Low tier (Provincial) | 2187.42 | (-659.52, 5034.36) | **0.02***** | **(<0.01, 0.04)** |
| **Job title (Reference: Lecturer or below)** | |  |  |  |  |
|  | Associate professor | **-2239.01***** | **(-3896.07, -581.95)** | **-0.01**** | **(-0.03, <0.01)** |
|  | Full professor | **-2979.83*** | **(-6394.35, 434.68)** | 0.01 | (-0.01, 0.03) |
| **Employment contract (Reference: Permanent)** | |  |  |  |  |
|  | Non-permanent | 516.13 | (-1402.37, 2434.62) | **0.01**** | **(<0.01, 0.02)** |
| **Too much pressure on teaching (Reference: Disagree)** | |  |  |  |  |
|  | Neutral | 172.81 | (-2253.43, 2599.06) | <0.01 | (-0.02, 0.01) |
|  | Agree | -1982.07 | (-4367.09, 402.95) | -0.01 | (-0.02, <0.01) |
| **Too much pressure on research (Reference: Disagree)** | |  |  |  |  |
|  | Neutral | -189.82 | (-4787.24, 4407.60) | <0.01 | (-0.03, 0.03) |
|  | Agree | -1040.27 | (-5634.90, 3554.36) | -0.02 | (-0.05, 0.01) |
| **Too much pressure on academic promotion (Reference: Disagree)** | |  |  |  |  |
|  | Neutral | **-3437.47*** | **(-7223.35, 348.42)** | 0.01 | (-0.01, 0.04) |
|  | Agree | **-3299.15*** | **(-6679.47, 81.16)** | 0.01 | (-0.01, 0.03) |
| **Box-Cox** | | **VAS** | | **utility index** | |
|  | **/theta** | 2.38**** | (1.70, 3.06) | 5.96**** | (4.84, 7.08) |

Note: * *P<0.1;* ** *P<0.05;* *** *P<0.01;* *****P<0.001*

| **Table S2. Predictors of VAS and utility index scores: results of two-level linear and Tobit regression models** | | | | | |
| --- | --- | --- | --- | --- | --- |
| **Variables** | | **Linear regression on VAS** | | **Tobit regression on utility index** | |
|  |  | **β** | **95% CI** | **β** | **95% CI** |
| **Fixed effects (Level 1)** | |  |  |  |  |
| **Gender (Reference: Male)** | |  |  |  |  |
|  | Female | 1.27 | (-1.22, 3.77) | 0.02 | (-0.02, 0.05) |
| **Age group (Years) (Reference: 21-30)** | |  |  |  |  |
|  | 31-35 | 0.41 | (-4.31, 5.13) | -0.01 | (-0.04, 0.01) |
|  | 36-40 | 0.18 | (-5.84, 6.20) | -0.01 | (-0.04, 0.03) |
| **Annual salary after-tax (Chinese Yuan) (Reference: ≤100000)** | |  |  |  |  |
|  | 100000-150000 | 0.36 | (-3.50, 4.23) | **0.01*** | **(<-0.01, 0.03)** |
|  | >150000 | 0.70 | (-4.77, 6.17) | **0.04**** | (**0.01, 0.07**) |
| **Regular physical activities (Reference: Yes)** | |  |  |  |  |
|  | No | **-2.31****** | (**-3.65**, **-0.97**) | **-0.02***** | (**-0.04, -0.01**) |
| **Regular sleeping (Reference: Yes)** | |  |  |  |  |
|  | No | -1.07 | (-2.41, 0.28) | **-0.02****** | (**-0.03, -0.02**) |
| **Regular meals (Reference: Yes)** | |  |  |  |  |
|  | No | **-6.21****** | (**-7.53**, **-4.89**) | -0.01 | (-0.04, 0.03) |
| **Actively participate in social activities (Reference: Disagree)** | |  |  |  |  |
|  | Neutral | 5.63 | (-1.63, 12.89) | 0.03 | (-0.02, 0.07) |
|  | Agree | **8.42***** | (**2.86**, **13.97**) | **0.05***** | (**0.02, 0.08**) |
| **Get along well with colleagues (Reference: Disagree)** | |  |  |  |  |
|  | Neutral | **11.88****** | (**5.88**, **17.89**) | -0.01 | (-0.10, 0.07) |
|  | Agree | **11.79****** | (**6.02**, **17.56**) | 0.01 | (-0.07, 0.09) |
| **Harmonious family relationship (Reference: Disagree)** | |  |  |  |  |
|  | Neutral | **16.81****** | (**9.70**, **23.92**) | 0.05 | (-0.07, 0.18) |
|  | Agree | **20.88****** | (**17.45**, **24.31**) | **0.07**** | (**0.00, 0.13**) |
| **Job title (Reference: Lecturer or below)** | |  |  |  |  |
|  | Associate professor | **-2.79***** | **(-5.80, 0.22)** | **-0.03****** | (**-0.04, 0.02**) |
|  | Full professor | -3.65 | (-9.95, 2.66) | 0.02 | (-0.03, 0.06) |
| **Employment contract (Reference: Permanent)** | |  |  |  |  |
|  | Non-permanent | 0.33 | (-4.74, 5.39) | 0.02 | (-0.01, 0.06) |
| **Too much pressure on teaching (Reference: Disagree)** | |  |  |  |  |
|  | Neutral | 0.35 | (-1.66, 2.35) | <0.01 | (-0.02, 0.02) |
|  | Agree | **-2.83****** | (**-4.18**, **-1.48**) | -0.01 | (-0.03, 0.01) |
| **Too much pressure on research (Reference: Disagree)** | |  |  |  |  |
|  | Neutral | -1.17 | (-5.78, 3.45) | 0.01 | (-0.08, 0.10) |
|  | Agree | -2.33 | (-7.43, 2.76) | -0.03 | (-0.09, 0.03) |
| **Too much pressure on academic promotion (Reference: Disagree)** | |  |  |  |  |
|  | Neutral | **-5.19***** | (**-8.48**, **-1.89**) | 0.02 | (-0.01, 0.04) |
|  | Agree | **-4.74****** | (**-6.32**, **-3.17**) | **0.03**** | (**>0.00, 0.05**) |
| **Random effects (Level 2)†** | |  |  |  |  |
|  | Variance (intercept) | 4.09 | (0.87, 19.36) | <0.01 | (0.00, 0.13) |

Note: * *P<0.10*; ** *P<0.05*; ****P<0.01*; *****P<0.001*

† ICC was 0.05 (0.01, 0.21) for EQ-VAS and 0.03 (>0.00, 0.97) for EQ-5D index scores.

| **Table S3. Predictors of VAS and utility index scores transformed by Box-Cox: results of two-level linear and Tobit regression models** | | | | | |
| --- | --- | --- | --- | --- | --- |
| **Variables** | | **Linear regression on VAS transformed by Box-Cox,**  **(θ=2.5)** | | **Tobit regression on utility index transformed by Box-Cox**  **(θ=6.0)** | |
|  |  | **β** | **95% CI** | **β** | **95% CI** |
| **Fixed effects (Level 1)** | |  |  |  |  |
| **Gender (Reference: Male)** | |  |  |  |  |
|  | Female | 895.40 | (-995.35, 2786.16) | 0.01 | (-0.01, 0.03) |
| **Age group (Years) (Reference: 21-30)** | |  |  |  |  |
|  | 31-35 | 618.69 | (-2717.99, 3955.36) | >-0.01 | (-0.02, 0.01) |
|  | 36-40 | 307.14 | (-4025.00, 4639.27) | >-0.01 | (-0.02, 0.02) |
| **Annual salary after-tax (Chinese Yuan) (Reference: ≤100000)** | |  |  |  |  |
|  | 100000-150000 | 58.57 | (-3031.00, 3148.14) | <0.01 | (-0.01, 0.02) |
|  | >150000 | 387.36 | (-3473.36, 4248.08) | 0.01 | (-0.01, 0.04) |
| **Regular physical activities (Reference: Yes)** | |  |  |  |  |
|  | No | **-1435.81***** | **(-2288.53, -583.09)** | **-0.01**** | **(-0.02, -0.001)** |
| **Regular sleeping (Reference: Yes)** | |  |  |  |  |
|  | No | -833.80 | (-1833.52, 165.92) | **-0.01****** | **(-0.01, -0.01)** |
| **Regular meals (Reference: Yes)** | |  |  |  |  |
|  | No | **-4258.00****** | **(-5194.01, -3321.99)** | >-0.01 | (-0.02, 0.01) |
| **Actively participate in social activities (Reference: Disagree)** | |  |  |  |  |
|  | Neutral | 3640.96 | (-741.88, 8023.79) | 0.01 | (-0.01, 0.03) |
|  | Agree | **5773.00***** | **(2128.32, 9417.68)** | **0.02****** | **(0.01, 0.03)** |
| **Get along well with colleagues (Reference: Disagree)** | |  |  |  |  |
|  | Neutral | **7601.37****** | **(4384.83, 10817.90)** | 0.01 | (-0.03, 0.04) |
|  | Agree | **7554.47****** | **(3994.47, 11114.47)** | <0.01 | (-0.03, 0.03) |
| **Harmonious family relationship (Reference: Disagree)** | |  |  |  |  |
|  | Neutral | **10008.80****** | **(5691.62, 14325.99)** | 0.03 | (-0.02, 0.08) |
|  | Agree | **12428.09****** | **(10095.33, 14760.86)** | **0.04**** | **(0.01, 0.07)** |
| **Job title (Reference: Lecturer or below)** | |  |  |  |  |
|  | Associate professor | **-2184.94**** | **(-4174.10, -195.77)** | **-0.01**** | **(-0.03, -0.002)** |
|  | Full professor | -2930.07 | (-6754.43, 894.29) | 0.01 | (-0.01, 0.03) |
| **Employment contract (Reference: Permanent)** | |  |  |  |  |
|  | Non-permanent | 357.15 | (-3254.05, 3968.34) | 0.01 | (-0.01, 0.03) |
| **Too much pressure on teaching (Reference: Disagree)** | |  |  |  |  |
|  | Neutral | 146.92 | (-1587.16, 1881.00) | >-0.01 | (-0.02, 0.02) |
|  | Agree | **-1966.82***** | **(-3296.48, -637.15)** | -0.01 | (-0.02, 0.01) |
| **Too much pressure on research (Reference: Disagree)** | |  |  |  |  |
|  | Neutral | -11.27 | (-3408.20, 3385.65) | <0.01 | (-0.05, 0.05) |
|  | Agree | -908.98 | (-3886.68, 2068.73) | -0.02 | (-0.05, 0.02) |
| **Too much pressure on academic promotion (Reference: Disagree)** | |  |  |  |  |
|  | Neutral | **-3540.18****** | **(-5522.17, -1558.19)** | 0.01 | (-0.01, 0.03) |
|  | Agree | **-3392.12****** | **(-4751.96, -2032.28)** | **0.01**** | **(<0.01, 0.02)** |
| **Random effects (Level 2)†** | |  |  |  |  |
|  | Variance (intercept) | 1924506 | (428238, 8648746) | <0.01 | (<0.01, 0.16) |
| **Box-Cox** | | **VAS** | | **utility index** | |
|  | **/theta** | 2.38**** | (1.70, 3.06) | 5.96**** | (4.84, 7.08) |

Note: * *P<0.10*; ** *P<0.05*; ****P<0.01*; *****P<0.001*

† ICC was 0.05 (0.01, 0.21) for Box-Cox transformed EQ-VAS and 0.03 (>0.00, 0.97) for Box-Cox transformed EQ-5D index scores.
